# Demographic and Clinical Determinants of Acute Bronchiolitis Outcomes in a Tertiary Pediatric Hospital in Dubai, United Arab Emirates

**DOI:** 10.7759/cureus.112747

**Published:** 2026-07-15

**Authors:** Hadi Helali, Arshiya Hashmath, Batool Khan, Fatima Mazahir

**Affiliations:** 1 Pediatric Neurology, Al Jalila Children's Specialty Hospital, Dubai, ARE; 2 Pediatric Neurology, Mohammed Bin Rashid University of Medicine and Health Sciences, Dubai, ARE; 3 Pediatric Medicine, Mohammed Bin Rashid University of Medicine and Health Sciences, Dubai, ARE; 4 Pediatric Hospital Medicine, Al Jalila Children's Specialty Hospital, Dubai, ARE

**Keywords:** bronchiolitis, hospital medicine, pediatrics, public health, respiratory syncytial virus, respiratory tract infection

## Abstract

Background: Bronchiolitis is a lower respiratory tract infection that affects children under two years of age and is considered a cause of increased morbidity and healthcare costs, especially during the winter season. Despite its global burden, evidence on how local demographic or clinical factors influence outcomes in Middle Eastern populations remains limited.

Objectives: This study aims to examine the descriptive epidemiology and different demographic and clinical characteristics of patients admitted with bronchiolitis, and to evaluate exploratory associations between these characteristics and various clinical outcomes, including duration of hospital admission, need for pediatric intensive care unit (PICU) admission, and need for invasive oxygen supplementation.

Methods: This retrospective study included all infants aged 1-24 months who were admitted with bronchiolitis between October 1, 2022, and March 31, 2023. Electronic records from Cerner and Salama (Epic) were screened. Variables extracted included age at presentation, sex, nationality, gestational age, feeding type, family history of atopy, viral PCR results, initial oxygen saturation, and therapies received. Primary outcomes were PICU admission, maximum oxygen support, and length of stay. Associations were assessed using chi‑square or Fisher’s exact tests for categorical variables and Mann-Whitney U tests for continuous variables, with significance set at p < 0.05.

Results: Of 1,804 screened encounters, 232 patients were eligible for the study. The mean (± SD) age was 6.6 ± 5.2 months, and 138 patients (59.5%) were male. Respiratory syncytial virus (RSV) was detected in 101 patients (43.5%), rhinovirus/enterovirus in 29 patients (12.5%), and mixed/other viruses in 53 patients (22.8%). A high-flow nasal cannula was used in 87 cases (37.5%), but only 14 patients (6.1%) required PICU admission. None of the demographic or clinical factors showed a statistically significant effect on the chance of PICU admission or oxygen requirement. A family history of atopy was the only factor associated with a shorter length of stay (Mann-Whitney U = 2503.5; p = 0.008). Steroid therapy was more frequently administered to infants requiring greater oxygen support (chi-square = 13.971; p < 0.001), but did not shorten hospital stays or reduce PICU admissions.

Conclusions: In this cohort from the United Arab Emirates, bronchiolitis outcomes were largely independent of demographic factors, viral etiology, or corticosteroid use. Adherence to international guidelines, which advocate for supportive care and discourage the routine use of glucocorticoids or bronchodilators, is likely to improve resource utilization without compromising outcomes.

## Introduction

Bronchiolitis is a lower respiratory tract infection that primarily affects infants and young children, with respiratory syncytial virus (RSV) being the most common causative agent, responsible for approximately 70% of cases [1]. The disease is characterized by inflammation and obstruction of the small airways in the lungs, leading to respiratory distress marked by wheezing, tachypnoea, and often hypoxia. Globally, bronchiolitis represents a substantial disease burden, leading to around 3.6 million hospitalizations and up to 118,000 deaths [2]. It has a notable peak during the winter months in temperate regions [3]. While bronchiolitis can be caused by other viruses such as rhinovirus, influenza, and parainfluenza, RSV bronchiolitis significantly contributes to the global disease burden, with approximately 33 million cases annually [2,4].

Clinically, the diagnosis is made based on characteristic symptoms and physical examination, although viral testing may be used in more severe cases or to guide specific treatment decisions. Currently, the management of bronchiolitis remains largely supportive, with treatments focusing on oxygen therapy, hydration, and nasal suctioning to clear the airways of mucus. There is limited evidence supporting the use of bronchodilators, corticosteroids, or antibiotics in routine cases; however, these therapies are sometimes employed in the hope of alleviating symptoms or preventing complications [5]. Preventive measures are crucial, particularly for high-risk infants, such as those born prematurely or with underlying cardiovascular or pulmonary conditions. In this context, palivizumab, a monoclonal antibody targeting RSV, has been used to reduce the risk of severe RSV infection in high-risk infants, though its high cost limits widespread use [6]. Research efforts are increasingly focused on developing a safe and effective RSV vaccine, with recent trials showing promising results in reducing RSV-related morbidity [7]. Preventive strategies have recently expanded to include maternal vaccination with the bivalent RSVpreF vaccine and infant immunization with the monoclonal antibody nirsevimab, both of which are recommended to prevent RSV infection during the first year of life. Early estimates suggest that nirsevimab reduces RSV-associated hospitalization by approximately 90% [8,9].

Despite these advances, bronchiolitis continues to be a major challenge for clinicians, and further research is needed to improve prevention, treatment, and outcomes for affected children. Studies from North America and Europe have extensively described the epidemiology of bronchiolitis and predictors of severe disease; however, data from the Middle East remain limited, except for notably a study from Saudi Arabia and another from Al Ain, United Arab Emirates. Understanding local demographic and clinical factors is essential to optimize resource allocation and ensure evidence‑based care. The present study, therefore, aims to examine all infants admitted to the pediatric tertiary hospital in Dubai, UAE, with bronchiolitis during one RSV season and explore the associations between patient characteristics and treatment outcomes.

## Materials and methods

Study design and setting

This was a retrospective cohort study conducted at Al Jalila Children’s Specialty Hospital. The hospital serves both Emirati citizens and expatriate families. The study period spanned one winter season, from October 1, 2022, to March 31, 2023. This was selected to capture the peak winter season, which coincides with the highest bronchiolitis activity in the United Arab Emirates. During this season, an increase in the number of hospitalized bronchiolitis patients receiving steroids to prevent PICU admission was also observed. All eligible pediatric patients presenting to the hospital between October 2022 and March 2023 were included. The Institutional Review Board (IRB) of Mohammed Bin Rashid University of Medicine and Health Sciences approved the study (MBRU IRB‑2023‑155), and the Strengthening the Reporting of Observational Studies in Epidemiology (STROBE) guideline was followed in reporting [10].

Participants and eligibility criteria

All infants aged 1-24 months admitted and discharged with a primary diagnosis of bronchiolitis during the study period were eligible. The bronchiolitis diagnosis was based on the physicians’ clinical assessment documented in the electronic record. Exclusion criteria were (1) age <1 month or >24 months; (2) underlying cardiac, endocrine, renal, or neurological disease that might influence treatment decisions; (3) proven bacterial co‑infection at presentation; (4) concurrent diuretic therapy, as it may affect basic metabolic status; and (5) hospitalization outside Al Jalila Children’s Specialty Hospital. These criteria were chosen to create a homogeneous cohort and minimize confounding.

Data sources and variables

We screened electronic medical records using two platforms: Cerner (Oracle Health, Austin, TX, USA) and Epic (Epic Systems Corporation, Verona, WI, USA) (locally referred to as “Salama”). Admission lists were filtered to identify infants with both an admission and a discharge diagnosis of bronchiolitis. Patient charts were then reviewed to confirm eligibility. Data were abstracted into a password‑protected spreadsheet and included: (1) Demographic data: sex, nationality (Emirati vs. expatriate), gestational age (term vs. preterm), age at presentation (categorized as <6 months, 6-12 months, 12-18 months, 18-24 months), feeding type (exclusive breastfeeding, formula, mixed, or not documented), family history of atopy, and ethnicity of expatriate infants; (2) Clinical data: month of admission, respiratory viral PCR results, initial oxygen saturation (<90% vs. ≥90%), maximum oxygen support required (room air, nasal cannula, high‑flow nasal cannula (HFNC) or invasive ventilation), chest radiography performed, and treatments received (nebulized bronchodilator, corticosteroid or other therapy); (3) Outcomes: (i) PICU admission - admission to the pediatric intensive care unit (PICU) for any reason during the hospital stay; (ii) Maximum oxygen requirement - highest level of oxygen support required (categorized as none/room air, low‑flow oxygen, HFNC or invasive ventilation); (iii) Length of hospitalization - time from admission to discharge, measured in days; (4) Missing data was observed in several variables, most notably family history of atopy (26.3% missing) and feeding history (12.9% missing). To handle missingness, we utilized complete-case analysis (listwise deletion) for our primary statistical tests, as the missing data were assumed to be missing at random (MAR) based on documentation gaps rather than clinical severity. While multiple imputation was considered, the low frequency of our primary outcome (PICU admission, n=14) limited the robustness of complex imputation models for this specific outcome.

Statistical analysis

Data were analyzed using IBM SPSS Statistics for Windows, Version 28 (Released 2021; IBM Corp., Armonk, New York, United States). Continuous variables were summarized as means ± standard deviations if normally distributed or medians (interquartile range) if skewed. Categorical variables were reported as counts and percentages. Associations between categorical predictors and dichotomous outcomes (PICU admission or maximum oxygen category) were assessed using chi‑square tests or Fisher’s exact tests when expected cell counts were <5. For continuous outcomes such as length of stay, we used the Mann-Whitney U test to compare medians across groups. A two‑sided p < 0.05 was considered statistically significant.

## Results

Participants

**Figure 1:**
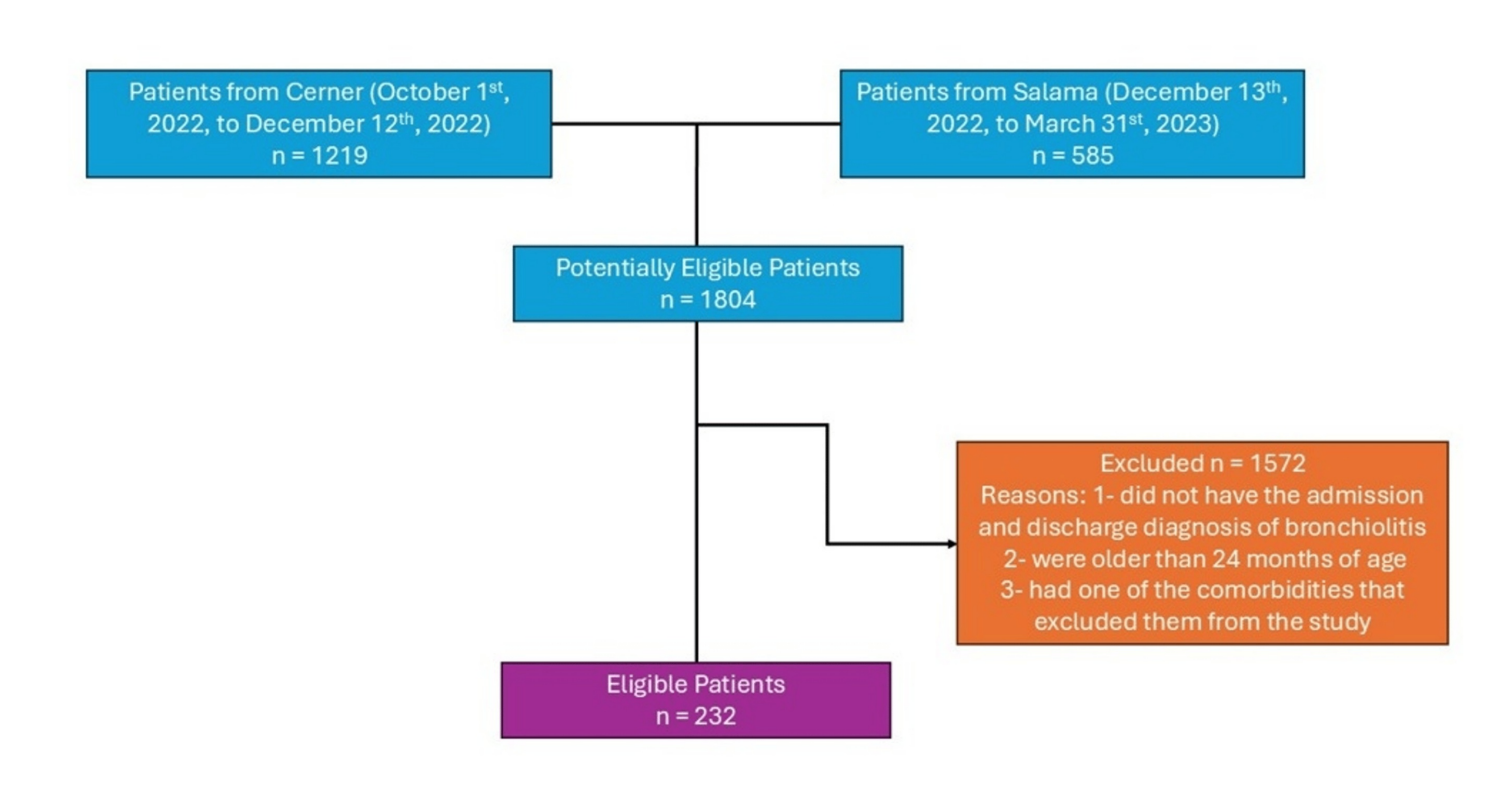
Flow Diagram of Patient Inclusion and Exclusion in the Study

Screening of 1804 admissions during the study period yielded 232 eligible infants (Figure 1). Most exclusions were due to missing diagnoses, age outside the range, or comorbidities specified in the exclusion criteria (Figure 1).

Demographic characteristics

**Figure 2:**
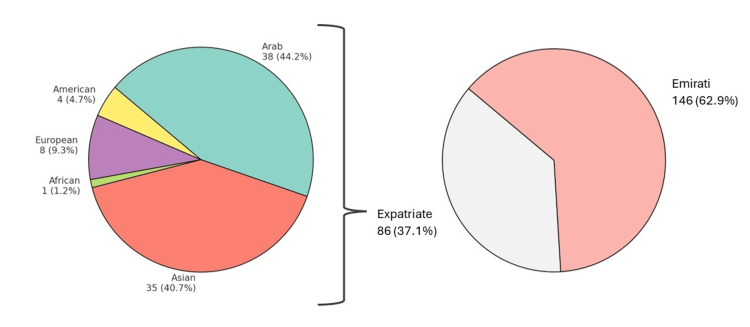
Ethnic Distribution of Study Participants Data are presented as N (%).

The mean age at admission was 6.6 ± 5.2 months (range 1-24 months). Age at presentation was distributed as follows: <6 months (63.4%), 6-12 months (20.3%), 12-18 months (9.9%), and 18-24 months (6.5%). Male patients constituted 59.5% (138/232) of the cohort, and 62.9% (146/232) were Emirati nationals (Figure 2). Overall, 63.8% were born at term, and 36.2% were preterm. Regarding feeding, 30.2% were exclusively breastfed, 20.7% were formula-fed, and 36.2% received mixed feeding, while the feeding history was missing for 12.9%. A family history of atopy was recorded in 25.4% and absent in 48.3%; this information was missing for 26.3%. Among expatriate infants, 44.2% were of Arab origin, 40.7% were Asian individuals, 9.3% were European individuals, 4.7% were American individuals, and 1.2% were African individuals (Table 1).

**Table 1:** Summary of Patients’ Demographic Characteristics Data are presented as N (%).

Characteristic	N	%
Gender		
Male	138	59.50
Female	94	40.50
Nationality		
Local (UAE)	146	62.90
Expatriate	86	37.10
Gestational age		
Full term	148	63.80
Preterm	84	36.20
Age at presentation		
<6 months	147	63.40
6-12 months	47	20.30
12-18 months	23	9.90
18-24 months	15	6.50
Feeding type		
Not mentioned	30	12.90
Breastfeeding	70	30.20
Formula	48	20.70
Mixed	84	36.20
Family history of atopy		
Not mentioned	61	26.30
Yes	59	25.40
No	112	48.30
Ethnicity of non-locals		
Arab individuals	38	44.20
Asian individuals	35	40.70
African individuals	1	1.20
European individuals	8	9.30
American individuals	4	4.70

Clinical characteristics

**Figure 3:**
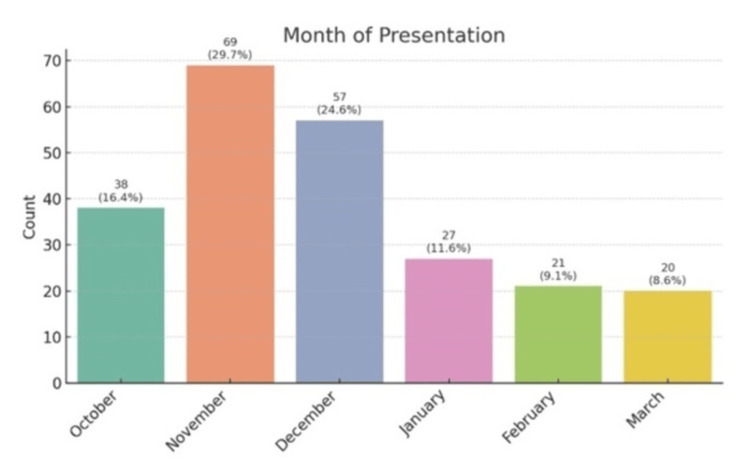
Distribution of Patient Admissions by Month Data are presented as N (%).

**Figure 4:**
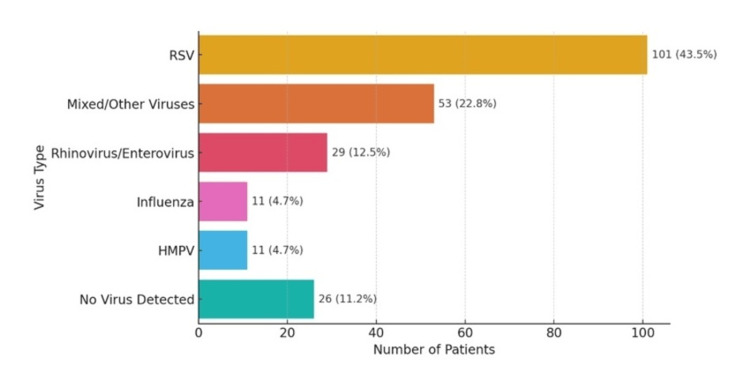
Viral Distribution During the 2022-2023 Winter Season Data are presented as N (%). RSV: respiratory syncytial virus; HMPV: human metapneumovirus

Admissions peaked in November (29.7%) and December (28.9%) (Figure 3), consistent with the winter season trend distribution of RSV in desert climates [2]. RSV was the most frequently identified virus, detected in 101 patients (43.5%), followed by mixed/other viruses in 53 patients (22.8%), and rhinovirus/enterovirus in 29 patients (12.5%). Influenza and human metapneumovirus (HMPV) were each detected in 11 patients (4.7%) (Figure 4). Most patients had an initial oxygen saturation of ≥90% (211 patients, 91.3%). A total of 87 patients (37.5%) required high-flow nasal cannula on admission. Only 14 patients (6.1%) required admission to the PICU, while the remaining 217 patients (93.9%) were managed in the general pediatric ward. A chest X-ray was performed in most patients (224 patients, 96.6%). Steroids (administered either intravenously or through inhaled nebulization) were given to 60 patients (25.9%). The median length of stay was 3 days (interquartile range, 2-5 days) and did not differ significantly by sex, nationality, or gestational age.

A subset of 14 patients (6.1%) required escalation of care to the PICU. Notably, 13 patients presented with an initial oxygen saturation below 90%, representing a strong clinical correlation with the eventual need for intensive care. All 14 patients in the PICU group required higher-tier support, including non-invasive or invasive mechanical ventilation. Steroid therapy was significantly more prevalent among these severely ill infants compared to those in the general ward (chi-square = 13.971; p < 0.001), though its administration did not statistically reduce the likelihood of PICU admission (chi-square = 0.160; p = 0.689). Consistent with the broader cohort, the requirement for PICU admission was not significantly influenced by demographic variables - including sex, nationality, or gestational age - nor by specific viral etiologies such as RSV (p > 0.05).

Association with treatment outcome

Neither demographic factors (sex, nationality, gestational age, feeding type, age at presentation, family history of atopy) nor viral aetiology significantly influenced the likelihood of PICU admission for non-invasive ventilation or invasive ventilation (all chi-square tests yielded p > 0.05). Similarly, there was no significant association between gestational age, nebulized bronchodilator use, or steroid therapy and hospitalization length. A family history of atopy was associated with a shorter mean hospital admission duration (3.17 days vs. 4.36 days; p = 0.008), while gestational age (p = 0.286), nebulization (p = 0.207), and steroid treatment (p = 0.554) did not significantly affect the duration.

Steroid administration was significantly more common among infants requiring higher-level oxygen support (HFNC, non-invasive, or invasive ventilation) (chi-square = 13.971; p < 0.001). However, steroid use did not shorten length of stay (Mann-Whitney U = 4898.5; p = 0.554) nor reduce the rate of PICU admission (chi-square = 0.160; p = 0.689). Preterm infants had higher odds of PICU admission, though not statistically significant (OR = 2.52, 95% CI (0.84-7.50), p = 0.088). Receiving steroids was not significantly associated with altered odds of PICU admission (OR = 0.76, 95% CI (0.21-2.83), p = 0.689). A family history of atopy showed a trend toward lower odds of PICU admission, but it did not reach statistical significance (OR = 0.20, 95% CI (0.02-1.62), p = 0.097). Receiving nebulization was not associated with PICU admission (OR = 0.57, 95% CI (0.15-2.10), p = 0.397). None of the 232 eligible infants in this specific cohort were documented as having received palivizumab prior to admission.

## Discussion

Summary of major findings

This study evaluated the demographic and clinical profile of infants hospitalized with bronchiolitis in Dubai and examined associations with key outcomes. Overall, 6.1% of infants required PICU admission, with a median length of stay of three days. Neither demographic characteristics nor viral etiology influenced the likelihood of PICU admission or requirement for high‑level oxygen support. Family history of atopy was associated with a modestly shorter hospitalization, and although steroids were more often used in infants requiring greater oxygen support, this therapy did not shorten length of stay or reduce PICU admission. These findings suggest that most infants admitted with bronchiolitis, even in a tertiary care setting, have a favorable clinical course and that demographic differences (sex, nationality, gestational age) do not meaningfully influence outcomes. The study did not detect significant associations between demographic factors and clinical outcomes. This lack of significance should not be interpreted as definitive evidence of independence; rather, it may be due to insufficient statistical power, the homogeneous nature of the cohort under strict exclusion criteria, or unmeasured confounding variables.

Comparison with previous studies

A study by Rao et al. (2023), conducted in Colorado, USA, showed a trend similar to that of our patient, with RSV as the most prevalent virus detected among bronchiolitis patients. On the other hand, they had a higher number of patients with COVID-19 infection leading to bronchiolitis than in our study. A total of 27.3% of their patients required PICU admission, while only 6.1% of our patients were managed there [11].

Another study by Al Shibli et al. (2021) conducted in Al Ain, UAE, showed similar demographic findings to our patient cohort (majority male, mean age around six months). It also showed a similar trend in chest X-ray utilization, but to a lesser degree (80% in their patients vs. 96.6% in ours). The PICU admission rate was closer to our number (11% vs. 6.1%), but a lower percentage of their patients received bronchodilator and oxygen therapy (42% vs. 59.5%, respectively). The mean duration of hospital admission was three days, which is similar to our data [12]. The notably high preterm rate (36.2%) observed in this cohort requires contextualization. This high prevalence is likely attributable to referral patterns to our tertiary care center rather than a direct reflection of the broader epidemiology of preterm birth in the UAE. Furthermore, variations in documentation and the precise definition of gestational age <37 weeks may have contributed to this finding.

A study by Haskell et al. (2021) highlighted the need to adhere to international standards in the management of bronchiolitis. This includes, but is not limited to, avoiding exposure of children to chest X-rays when the diagnosis is clinically clear, and avoiding steroid nebulization in patients with straightforward bronchiolitis, a practice that is still employed, as shown in our data [13].

While our single-season snapshot demonstrated a low PICU admission rate and no mortality, longitudinal studies from other regions highlight the dynamic nature of bronchiolitis outcomes over time. For instance, a 14-year national trend study in Canada by Mahant et al. (2021) observed escalating trends in PICU admissions, associated costs, and mortality [14]. Although their long-term, national design precludes a direct comparison with our single-center cohort, their findings underscore a critical gap in the literature: the necessity for long-term, multi-center surveillance in the Middle East to determine if similar shifts in bronchiolitis severity and healthcare utilization are occurring in this region.

A notable finding in this cohort was the high preterm birth rate (36.2%), which was significantly above the typical global prevalence [13,14]. Despite this, gestational age did not emerge as a significant predictor for PICU admission or increased length of stay in our analysis (p > 0.05). This discrepancy with some international literature may be due to our exclusion of infants with significant cardiac or complex neurological comorbidities, who often constitute the highest-risk segment of the preterm population. Consequently, our results suggest that, for otherwise healthy infants with bronchiolitis, prematurity alone may not predict a more severe clinical course in this regional setting.

The observation of a shorter length of stay among infants with a family history of atopy is counterintuitive. Several possibilities may explain this finding. First, there may be confounding factors, such as atopic families seeking care earlier for milder disease, or clinicians discharging them sooner, anticipating a viral-triggered wheeze rather than true bronchiolitis. Second, misclassification may have occurred, where some of these infants actually experienced viral-triggered wheeze or early asthma rather than true bronchiolitis. Finally, given the multiple comparisons made in this study, this association could simply represent a type I error.

Clinical implications and guideline adherence

Importantly, our analysis found no evidence that steroids shortened hospital stay or reduced PICU admission. This supports international guidelines that recommend against routine use of systemic or nebulized corticosteroids for bronchiolitis. Evidence synthesis suggests that bronchodilators, racemic epinephrine, and glucocorticoids do not improve clinical outcomes in uncomplicated bronchiolitis. Although steroids were more frequently administered to infants requiring greater oxygen support, the unadjusted analysis provided in this study is uninformative regarding true steroid efficacy. Because sicker patients preferentially receive steroids, propensity score matching or multivariable adjustment would be required to robustly evaluate whether this therapy effectively shortens hospital stays or reduces PICU admissions. The high use of chest radiography and steroids in our cohort highlights opportunities to align local practice with evidence‑based guidelines. Educational interventions and the introduction of local clinical pathways may reduce unnecessary interventions and costs, as shown by randomized trials in other settings [13].

At the time of the study, palivizumab had been established as the standard of care for RSV prophylaxis in specific high-risk pediatric groups [9]. The absence of documented palivizumab use in our study reflects our exclusion criteria, which removed infants with the chronic conditions that typically qualify for such prophylaxis.

Future directions/preventive strategies

Our study also provides baseline regional data ahead of the widespread implementation of RSV preventive therapies. The maternal RSV vaccine and the long‑acting monoclonal antibody nirsevimab have recently been recommended to protect infants during their first RSV season. Early post-introduction data from the United States show that nirsevimab is approximately 90% effective at preventing RSV-associated hospitalization, while pooled trial data indicate approximately 81% efficacy against hospitalization. These interventions are expected to reduce the burden of bronchiolitis and may alter hospitalization patterns in the near future [9].

Strengths and limitations

This study benefits from a relatively large sample drawn from the only tertiary pediatric hospital in Dubai, allowing comprehensive capture of cases during one RSV season. However, the retrospective design carries inherent limitations, including missing or incomplete documentation (e.g., feeding type, family history of atopy) and potential misclassification of variables. Our findings reflect practice in a single public hospital and may not be generalizable to private institutions. Due to sample size constraints, multivariable regression analyses were not performed; therefore, the possibility of residual confounding cannot be ruled out. The study window (October 2022 to March 2023) does not represent the full annual viral cycle. Although the univariate analysis was robust, the low frequency of PICU admission (n = 14) limited the statistical power required for a stable multivariable regression model. The study was conducted prior to the establishment of hospital-wide guidelines for the management of bronchiolitis, which explains the differences with internationally recognized guidelines and the variability in management between different physicians. We acknowledge that excluding high-risk groups (e.g., cardiac patients) limits the baseline scope and that future studies should include them to better evaluate the impact of new immunization strategies. In the future, a larger-scale, prospective, randomized controlled trial should be conducted to properly assess the demographic and clinical factors that influence treatment outcome.

A significant limitation of this study is the limited statistical power regarding the PICU admission outcome. With a total cohort of 232 patients but only 14 PICU admission events (6.1%), the study did not meet the standard epidemiological threshold of 10 events per variable (EPV) required to adequately power a multivariable logistic regression model. A post hoc power analysis indicates that, given our event rate, our sample was only sufficiently powered to detect unusually large effect sizes (odds ratios > 3.5). Consequently, the lack of statistically significant predictors for PICU admission should be interpreted with caution, as it may represent a type II error rather than true clinical independence. To mitigate this, future multi-center studies with larger sample sizes are required.

## Conclusions

The diagnosis of bronchiolitis in pediatric patients remains primarily clinical, and the disease is usually self-limited, requiring primarily supportive treatment. While our descriptive findings broadly align with the expected outcomes of standard care, the study design limits direct testing of adherence to international guidelines. Emerging preventive strategies, such as maternal RSV vaccination and the monoclonal antibody nirsevimab, may further reduce the burden of severe bronchiolitis in the region; however, appropriately powered prospective studies are needed to identify robust predictors of clinical outcomes.
